# Choline Chloride-Based Deep Eutectic Solvents as Green Effective Medium for Quaternization Reactions

**DOI:** 10.3390/molecules27217429

**Published:** 2022-11-01

**Authors:** Valentina Bušić, Maja Molnar, Vice Tomičić, Dalia Božanović, Igor Jerković, Dajana Gašo-Sokač

**Affiliations:** 1 Department of Applied Chemistry and Ecology, Faculty of Food Technology Osijek, Josip Juraj Strossmayer University of Osijek, 31000 Osijek, Croatia; 2 Faculty of Chemistry and Technology, University of Split, 21000 Split, Croatia

**Keywords:** isonicotinamide salts, deep eutectic solvents, microwave synthesis, ultrasound synthesis

## Abstract

The Menshutkin reaction represents the alkylation of tertiary amines by alkyl halide where the reactants are neutral and the products, quaternary ammonium salts, are two ions with opposite signs. The most commonly used organic solvents in quaternization reactions are volatile organic solvents (VOSs), namely acetone, anhydrous benzene, dry dichloromethane (DCM), dimethylformamide (DMF) and acetonitrile (ACN). The purpose of this work was to examine eutectic solvents as a “greener” alternative to conventional solvents so that quaternization reactions take place in accordance with the principles of green chemistry. Herein, sixteen eutectic solvents were used as replacements for volatile organic ones in quaternization reactions of isonicotinamide with substituted phenacyl bromides. The reactions were carried out at 80 °C by three synthetic approaches: conventional (4–6 h), microwave (20 min) and ultrasound (3 h). Microwave-assisted organic reactions produced the highest yields, where in several reactions, the yield was almost quantitative. The most suitable eutectic solvents were based on choline chloride (ChCl) as the hydrogen bond acceptor (HBA) and glycerol, oxalic or levulinic acid as hydrogen bond donors (HBDs). The benefits of these three deep eutectic solvents (DESs) as a medium for quaternization reactions are the simplicity of their preparation for large-scale production, with inexpensive, available and nontoxic starting materials, as well as their biodegradability.

## 1. Introduction

**Figure 1:**
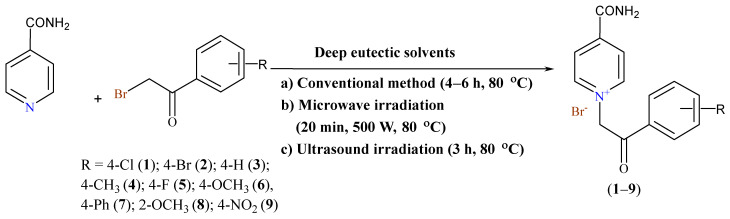
Synthesis of quaternary isonicotinamide salts from isonicotinamide and substituted phenacyl bromides in DESs.

Environmental pollution is mainly caused by the release of various chemicals into the atmosphere, which has increased drastically in the past several decades. It is the responsibility of scientists to design chemical products and processes that minimize or eliminate the use or production of substances hazardous to living beings and the environment. Solvents provide mass and energy transfer, and without them, many reactions cannot proceed. Unfortunately, solvents are also major contributors to the overall toxicity profile and thus represent the majority of materials of concern. They also contribute the greatest concern for process safety issues because they are flammable and volatile, even explosive. The Menshutkin reaction represents the reaction by which tertiary amines are converted to quaternary salts. In this *S_N_2* reaction, the neutral reactants are converted to charged products. In earlier research, toxic, volatile and hazard solvents such as acetone [1], anhydrous benzene [2], dry dichloromethane (DCM) [3], dimethylformamide (DMF) and acetonitrile (ACN) [4] were used for the quaternization reaction using the conventional method. According to 12 principles of green chemistry [5], we tried to focus our research on the use of safer and more environmentally friendly solvents. Deep eutectic solvents (DESs) can be useful in reducing the organic solvent waste in coming years. They became interesting due to their applicability as green solvents in the synthesis of pyridinium compounds [6,7,8,9,10,11]. DESs have found many useful applications in organic synthesis, which has resulted in numerous reviews in recent years [12,13,14,15,16]. DESs are mixtures formed from Lewis or Brønsted acids and bases containing various anionic and/or cationic species. They are usually obtained by the complexation of a quaternary ammonium salt with a metal salt or hydrogen bond donor HBD. The melting point of the eutectic solvent itself is lower than the melting point of its individual components due to delocalization of the charge within the hydrogen bond [17]. Recently, our research group successfully performed the first quaternization of nicotinamide with substituted 2-bromoacetophenone in sixteen choline chloride-based DESs [18]. In this research, we continue with the challenges of quaternization on the isonicotinamide (INA) moiety, shown in Figure 1. Choline chloride (ChCl) is used due to its low toxicity, biodegradability and low cost. The low cytotoxicity of some choline chloride-based DESs that we used in this research has been proven in other studies [19,20,21,22]. 

In our previous work, we prepared quaternary isonicotinamide salts under MW irradiation in two different solvents, EtOH and acetone [23]. The purpose of this study is to investigate whether classical organic solvents for the quaternization reaction of INA with phenacyl bromides can be substituted by eutectic solvents. Furthermore, the aim is to determine which eutectic solvent will be the most effective. Three different methods of synthesis are used: conventional, ultrasound and microwave irradiation. Based on the obtained results, we can conclude which of the three methods is the most efficient for the quaternization reaction in DESs. 

The physicochemical properties of DESs are also affected by the molar ratio of HBA and HBD, the purity of HBA and HBD, temperature, water content and the method of preparation (Table 1). The density is dependent on the packing and molecular organization of the DESs. They are composed of holes and empty vacancies which govern the density behavior. Mostly, the densities decrease with increasing temperature. The literature reveals that most DES densities are higher than the density of water, between 1.0 and 1.35 g cm^−3^ at 298.15 K [24]. DESs composed of ChCl and various acids such as HBDs were reported between 1.0 and 1.6 g cm^−3^ [25]. Most of the DESs exhibit a relatively high viscosity at room temperature (>0.1 Pa s) compared to molecular solvents. Viscosity is related to the free volume and the probability of finding holes of suitable dimensions for the solvent molecules or ions to move into. It is also dependent on the size of the ions.

## 2. Results and Discussion

Quaternization reactions were performed by the conventional method (as shown in Table 2), the ultrasound method (as shown in Table 3) and the microwave method (as shown in Table 4) in sixteen ChCl DESs. In the research, eutectic solvents of different molar ratios were used to gain insight into how the viscosity of solvents is affected during quaternization reactions. The lowest product yields were obtained by the conventional method: in 80% of reactions, the yield was 3–40%; in 14% of reactions, the yield was between 41% and 60%; and only 6% of reactions gave 61–96% yield. The suitable DESs for quaternization by the conventional method were ChCl/glycerol (6–75%), with the highest yield obtained for compound **9** (75%); ChCl/oxalic acid (13–96%), with the highest yield obtained for compound **7** (96%); and ChCl/levulinic acid (15–75%), with the highest yield obtained for compound **7** (75%). 

By the use of the ultrasound method, it is possible to carry out various homogeneous and heterogeneous organic reactions under milder conditions and in higher yields than by classical methods. In recent years, the ultrasound method has been intensively researched as a promising green technique in several organic transformations. In our research, 65% of reactions performed by the ultrasound method exhibited product yield of 4–41%, 24% of reactions exhibited yield between 41% and 60% and 11% of reactions exhibited yield of more than 60% (Table 3). The best yields were in DES ChCl/glycerol (25–84%), with the highest yield obtained for compound **3** (84%); in DES ChCl/oxalic acid (34–97%), with the highest yield obtained for compound **7** (97%); and in DES ChCl (38–94%), with the highest yield obtained for compound **3** (94%). First, the reactions were carried out at room temperature, but the formation of the product did not occur even after 24 h. By optimizing the reaction conditions, the highest yields were obtained at 80 °C. 

The ultrasound method in the present research also showed the lowest yields in two sugar-based eutectic solvents (glucose and fructose). From almost all reaction mixtures, products were not isolated from these eutectic solvents, but they were identified by TLC. Higher yields were achieved for DESs based on their alcohols (xylitol, sorbitol and glycerol) (Table 3). 

The microwave method showed that only 25% of reactions gave a yield of 12–40%, 26% gave a yield of 41–60%, and 50% of reactions obtained a yield higher than 60% (Table 4). The most appropriate DESs were ChCl/levulinic acid (65–95%), with the highest yield obtained for compound **3**; ChCl/urea (62–93%), with the highest yield obtained for compound **7**; and ChCl/oxalic acid (the yields were 54–98%). The highest yield was achieved for compound **4** (98%) in DES ChCl/oxalic acid. 

From the above results, it is evident that higher yields in the quaternization reaction were obtained in less viscous eutectic solvents.

DESs based on sugars (glucose, fructose) as a hydrogen bond donor are the most viscous and have the highest density. They were not suitable for the implementation of the quaternization reaction, which was evident from the low yields.

It has been proven that acid-based eutectic solvents are the most polar, so this is one of the possible reasons for obtaining the highest yields in the DESs choline chloride:oxalic and choline chloride:levulinic acid.

We assume that syntheses under the influence of microwave radiation gave the highest yields due to the polarity of the eutectic solvents as well as the starting materials themselves. Radiation is selectively absorbed by polar eutectic solvents, a characteristic that leads to selective heating profiles. The presence of a polar solvent, reagent or support in the reaction media leads to strong coupling with the radiation. This fact is particularly important in heterogeneous systems where it could also generate microscopic hot spots or selective heating [37].

In general, eutectic solvents have been proven to be suitable alternatives to conventional solvents. Since the lowest yields were obtained for fruit sugar-based eutectic solvents (glucose and fructose), there remains room for research on some other sugars and sugar alcohols. An exception in DES based on sugar alcohols is choline chloride:glycerol, which has proven to be a suitable alternative medium for the quaternization reaction.

**Figure 2:**
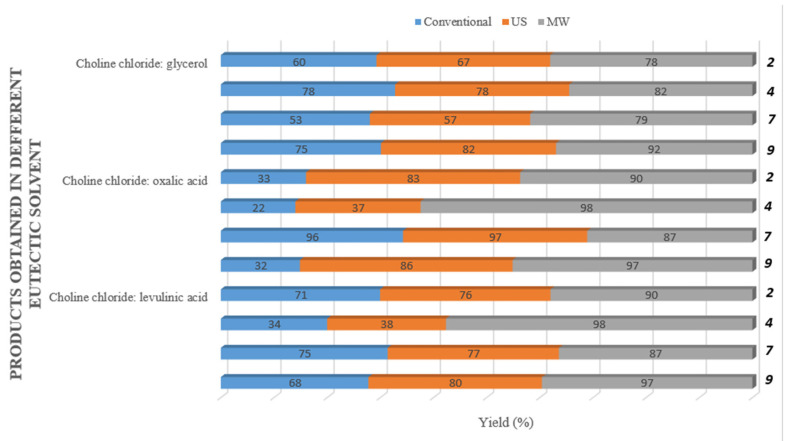
Yields (%) of the compounds **2**, **4**, **7** and **9** obtained by conventional (blue), ultrasonic (orange) and microwave (grey) methods in three eutectic solvents (ChCl/glycerol, ChCl/oxalic acid and ChCl/levulinic acid). The yields depend on the chosen eutectic solvent, but there was a significant increase when microwave irradiation was used.

The results from the present study show that the yields depend on the chosen solvent, but a significant yield increase was noticed when microwave irradiation was used (Figure 2).

Of the three examined methods, the microwave method proved to be the most effective. In summary, we have developed a straightforward, green and efficient protocol for the conventional, microwave and ultrasonic synthesis of quaternary salts of isonicotinamide in deep eutectic solvents. These reactions constitute a novel application of such reagents in heterocyclic synthesis. The procedures involve easily available starting materials and require remarkably short reaction times. They afford the desired products in adequate to high yields and avoid the use of volatile organic solvents in quaternization. To our knowledge, this is the first study of quaternization reactions of isonicotinamide quaternary salts performed in DESs according to the principles of green chemistry.

## 3. Experimental

### 3.1. Materials and Methods

Microwave-assisted synthesis was carried out in a Milestone flexi WAVE (Milestone, Sorisole, BG, Italy) microwave system, outfitted with a rotating carousel with 15 positions for PTFE high-pressure vessels. An ultrasonic (US) bath (BANDELIN electronic GmbH & Co. KG, Berlin, Germany, DT 510 H, frequency 35 Hz, nominal output 160 W, temperature 20–80 °C, power 400 W) was used for ultrasound synthesis. Thin-layer chromatography was performed on fluorescent silica gel plates F254 (Merc, Darmstadt, Germany) under UV light (254 and 365 nm) using chloroform:methanol (6:1.5 *v*/*v*) to monitor the progress of the reaction. The solvents and reagents were purchased from Merck (Darmstadt, Germany) and were used without further purification: urea 98.5%, *N*-methylurea 97%, thiourea 99%, D-(+)-glucose anhydrous, D-(−)-fructose 98.5%, xylitol 99%, D-sorbitol ≥ 98%, glycerol anhydrous, acetamide 99%, malic acid ≥ 98%, citric acid anhydrous, malonic acid 99%, oxalic acid 99.5%, L-(+)-lactic acid 98%, levulinic acid 98%, *trans*-cinnamic acid 99%. For quaternization reactions, we used isonicotiamide (Merck Group, 99%) and substituted phenacyl bromides from Aros Organics: 2-bromo-4-chloroacetophenon 98%, 2,4′-dibromoacetophenon 98%, 2-bromoacetophenon 98%, 2-bromo-4-methylacetophenon 97%, 2-bromo-4-floroacetophenon 97%, 2-bromo-4-methoxyacetophenon 98%, 2-bromo-4-phenylacetophenon 98%, 2-bromo-4-methoxyacetophenon 98%, 2-bromo-4-nitroacetophenon 95%.

The structures of prepared compounds were identified on the basis of ^1^H and ^13^C NMR spectra, IR spectra and elemental analyses in our previous work [23], where syntheses in classical organic solvents were performed.

### 3.2. Preparation of Deep Eutectic Solvents

The preparation of deep eutectic solvents was carried out by the mixing and heating at 80 °C of ChCl and various HBDs on a magnetic stirrer for a certain time depending on the HBDs. During stirring and heating, a stable homogeneous liquid was formed, which was cooled and used without further purification in quaternization reactions. Different DESs were prepared according to already known procedures [27,28,38].

ChCl was dried at 65 °C for 24 h to remove any possible moisture. DES ChCl:glycerol (1:2 molar ratio) was prepared by heating glycerol first up to 80 °C and then adding ChCl [39]. Fruit sugar-based deep eutectic solvents were prepared according to Hayan et al. [40].

### 3.3. Quaternization Reaction

The equimolar mixture of INA (1.2 mmol, 0.145 g) and substituted phenacyl bromides was dissolved in DES (molar ratios of INA and ChCl = 1:10), and the reaction mixture was subjected to three different synthetic methods.

#### 3.3.1. Conventional Method

In the conventional method, the reaction mixture was mixed on a magnetic stirrer for 4–6 h at 80 °C.

#### 3.3.2. Microwave Method

The reaction mixture was irradiated for 20 min at 80 °C at 500 W. 

#### 3.3.3. Ultrasonic Method

In the ultrasound method, the reaction mixture was sonicated at 80 °C in the US bath.

The reaction progress was monitored by thin-layer chromatography. After the reaction was completed, absolute ethanol (5 mL) was added into the reaction mixture, and the product was precipitated for the next 24 h. The crude product was filtered and purified by recrystallization from the appropriate solvent (methanol or mixture of ethanol and ethyl acetate 1:1 *v*/*v*).

## 4. Conclusions

In summary, a straightforward, green and efficient protocol for the conventional, microwave-assisted and ultrasonic synthesis of quaternary salts of isonicotinamide in deep eutectic solvents was developed. Among the procedures studied, the use of ChCl:oxalic acid as DES under microwave conditions stands out as the superior method with respect to the yields and short reaction times. A possible reason for this is the polarity of these DESs and the starting materials. Comparing the viscosity of eutectic solvents with the reaction yields, it is evident that less viscous eutectic solvents are more suitable for the quaternization reaction. This reaction constitutes a novel application of such reagents in heterocyclic synthesis. The procedures involve easily available starting materials and require remarkably short reaction times. They afford the desired products in adequate to high yields and avoid the use of volatile organic solvents in quaternization. To our knowledge, this is the first study of the quaternization reaction of isonicotinamide quaternary salts performed in DES according to the principles of green chemistry.

## Figures and Tables

**Table 1:** Physical parameters of the tested DESs.

HBA	HBD	Molar Ratio ChCl/HBD	Water Content (%)	Viscosity (Pa s)	Conductivity (μS cm^−1^)	Density (ρ) (g cm^−3^)	References
ChCl	Urea	1:2	1.89 ± 0.01	0.214 (30 °C)	1287	1.1879	[26]
ChCl	*N*-methylurea	1:3					[27]
ChCl	Thiourea	1:2		2.972 (35 °C)		1.36	[28]
ChCl	Glucose	1:1		34.400 (50 °C)			[29]
ChCl	Fructose	1:1				1.272	[30]
ChCl	Xylitol	1:1	1.21 ± 0.01	3.867 (30 °C)	172.6	1.2445	[31]
ChCl	Sorbitol	1:1	1.10 ± 0.02	13.736 (30 °C)	63.3	1.2794	[31]
ChCl	Glycerol	1:2	1.68 ± 0.01	0.177 (30 °C)	1647	1.18	[32,33]
ChCl	Acetamide	1:2	2.83 ± 0.02	0.127 (30 °C)	2710	1.09	[34]
ChCl	Malic acid	1:1	1.72 ± 0.01	11.475 (30 °C)	41.4	1.2796	[35]
ChCl	Citric acid	1:2					[34]
ChCl	Malonic acid	1:1	3.36 ± 0.01	0.616 (30 °C)	732	1.2112	[34]
ChCl	Oxalic acid	1:1	6.68 ± 0.02	0.089 (30 °C)	2350	1.2371	[34]
ChCl	Lactic acid	1:2				1.138	[36]
ChCl	Levulinic acid	1:2	2.55 ± 0.01	0.119 (30 °C)	1422	1.1320	[34]
ChCl	*Trans*-cinnamic acid	1:1				1.259	[29]

**Table 2:** Yields (**%**) of the conventional method for the synthesis of isonicotinamide quaternary salts (**1**–**9**) over 4–6 h at a temperature of 80 °C.

Entry	DES (ChCl:HBD)		Reaction Time	Yield (%)								
	HBD	Molar Ratio		1	2	3	4	5	6	7	8	9
1	Urea	1:2	4	3	12	18	37	44	46	48	32	71
2	*N*-methylurea	1:3	4	8	10	44	30	24	39	29	22	44
3	Thiourea	1:2	4	/ ^a^	14	17	15	22	25	37	31	32
4	Glucose	1:1	6	/	/	/	/	/	/	/	/	/
5	Fructose	1:1	6	/	/	/	/	/	/	/	/	/
6	Xylitol	1:1	6	/	/	/	/	/	/	/	/	/
7	Sorbitol	1:1	6	/	/	/	/	/	/	/	/	/
8	Glycerol	1:2	4	8	60	47	67	23	42	53	6	75
9	Acetamide	1:2	4	31	12	24	32	32	40	24	23	34
10	Malic acid	1:1	4	7	23	17	44	47	46	50	31	43
11	Citric acid	1:2	4	2	60	33	27	23	25	53	16	22
12	Malonic acid	1:1	4	24	8	30	10	16	44	47	10	64
13	Oxalic acid	1:1	4	26	33	13	22	30	41	96	35	32
14	Lactic acid	1:2	4	/	11	36	25	22	27	18	5	18
15	Levulinic acid	1:2	4	30	71	36	34	23	69	75	15	68
16	*Trans*-cinnamic acid	1:1	6	7	27	31	40	34	22	16	6	25

^a^ Products obtained in traces, not isolated.

**Table 3:** Yields (**%**) of the ultrasound method for the synthesis of isonicotinamide quaternary salts (**1**–**9**) over 3 h at a temperature of 80 °C.

Entry	DES (ChCl:HBD)		Yield (%)								
	HBD	Molar Ratio	1	2	3	4	5	6	7	8	9
1	Urea	1:2	7	14	28	55	57	60	52	11	53
2	*N*-methylurea	1:3	24	12	50	35	34	56	34	15	46
3	Thiourea	1:2	/ ^a^	14	21	30	33	34	40	20	57
4	Glucose	1:1	/	/	4	5	/	/	/	/	10
5	Fructose	1:1	/	/	4	3	/	/	/	/	6
6	Xylitol	1:1	/	18	13	11	20	23	12	18	22
7	Sorbitol	1:1	/	20	15	21	14	33	10	23	15
8	Glycerol	1:2	28	67	84	78	25	53	57	26	82
9	Acetamide	1:2	10	38	44	56	42	43	50	31	88
10	Malic acid	1:1	25	23	10	33	46	47	57	34	76
11	Citric acid	1:2	15	24	27	35	34	45	47	24	52
12	Malonic acid	1:1	10	23	48	10	34	44	40	29	55
13	Oxalic acid	1:1	34	83	49	37	48	62	97	41	86
14	Lactic acid	1:2	10	14	31	30	22	50	25	22	40
15	Levulinic acid	1:2	38	76	94	38	54	72	77	45	80
16	*Trans*-cinnamic acid	1:1	10	36	43	42	27	57	45	30	71

/ ^a^ Products obtained in traces, not isolated.

**Table 4:** Yields (**%**) of the microwave method for the synthesis of isonicotinamide quaternary salts (**1**–**9**) over 20 min at a temperature of 80 °C.

Entry	DES (ChCl:HBD)		Yield (%)								
	HBD	Molar Ratio	1	2	3	4	5	6	7	8	9
1	Urea	1:2	78	89	87	85	84	89	70	62	93
2	*N*-methylurea	1:3	59	36	78	40	34	44	62	65	67
3	Thiourea	1:2	66	56	69	47	55	37	61	44	58
4	Glucose	1:1	36	18	34	25	26	20	12	33	20
5	Fructose	1:1	22	28	43	30	24	19	26	48	21
6	Xylitol	1:1	32	30	41	47	26	13	37	27	29
7	Sorbitol	1:1	37	41	57	55	48	56	48	47	18
8	Glycerol	1:2	68	78	70	82	50	67	70	76	92
9	Acetamide	1:2	45	66	87	89	60	54	83	54	90
10	Malic acid	1:1	77	88	89	93	66	97	86	61	48
11	Citric acid	1:2	80	62	74	77	59	77	89	46	62
12	Malonic acid	1:1	89	80	80	72	50	77	88	58	67
13	Oxalic acid	1:1	96	90	87	98	78	80	87	54	97
14	Lactic acid	1:2	35	44	32	72	39	86	56	41	43
15	Levulinic acid	1:2	78	65	95	92	90	81	90	78	87
16	*Trans*-cinnamic acid	1:1	43	38	45	39	41	40	56	37	45

## Data Availability

Not applicable.

## Abbreviations

VOS	volatile organic species
DCM	dichloromethane
DMF	dimethylformamide
ACN	acetonitrile
DES	deep eutectic solvents
INA	isonicotinamide
ChCl	choline chloride
HBA	hydrogen bond acceptor
HBD	hydrogen bond donor
MW	microwave
US	ultrasound
EtOH	ethanol
